# Extra Supplement—The Nursing Mirror

**Published:** 1890-10-18

**Authors:** 


Being the Extra Nursing Supplement of "The Hospital" Newspaper.

All Contributions for this Supplement should be addressed to the Editor, The Hospital, 140, Strand, London, W.C., and should have the word "Nursing" plainly written in left-hand top corner of the envelope.

### En Passant.

EDINBURGH ROYAL INFIRMARY.—The managers of the infirmary have decided to proceed with the building of their Nurses' Home, notwithstanding the protest of the Dean of Guild Court. They considered the objections raised by the Court, and point out that in the original plans it was intended to have a three-storey pavilion on the proposed site, whereas the Nurses' Home is to be only 26 ft. high to the ridge of the roof. As to the objection to the building being quadrangular, it is pointed out that the space in the centre is 60 ft. square, and that the whole south front is to stand on stilts so as to allow the free passage of air. The managers also mention that since the infirmary was built the number of patients treated has increased by 60 per cent., and this necessarily implied a great increase in the number of nurses and servants, proper accommodation for whom is urgently required.

SHORT ITEMS.—One of Mrs. Levick's nurses is leaving Paris in November for India, to take up her post there as one of Lady Roberts's nursing sisters.—Nurse Hill and Nurse Hilda Garde are starting a home for strumous children in Oxfordshire; we wish them all success; such a home is badly needed.—Miss Stewart, matron of St. Bartholomew's, is one of the Stewarts of Appin.—In time of war the nursing staff in the field hospitals only numbers one orderly to seven patients.—Miss Reynolds lately gave a course of nursing lectures to the members of the Young Women's Christian Association at Kilkee.—Mrs. J. C. Mackenzie had a social gathering of nurses at the Morley Rooms lately, and addressed them on the subject of temperance.—Sister Ellinor and the sisters and probationers of the Red Cross Order were present at the opening of the winter session of Meath Hospital Medical School, when Dr. Smyly delivered the inaugural address to the students.

LECTURES.—The Gresham Lectures on Physic, delivered lately, dealt with the preservation of health, and it was delightful to see the guilty looks the many nurses present cast at one another when Dr. Symes Thompson inveighed against constant tea-drinking. According to Dr. Symes Thompson, the arrangement of meals is a fine art and deserves more thought and study. Bad cooking and bad teeth are frequent causes of disease. Be it remarked that nurses ought to pay special attention to their teeth, and never allow an unsound tooth to remain a day in their head. The last lecture of the course was most interesting, dealing strongly with the modern rage for hypnotism and for new remedies generally. In conclusion, Dr. Symes Thompson pronounced in favour of gymnastics and outdoor exercise as the best preservers of health. At the Nurses' Club on Friday, to hear Dr. Griffiths' lecture, there was a gathering which quite overflowed the small room, and the many nurses present were thoroughly repaid for coming by a most excellent lecture on the conditions of pregnancy which need the presence of a doctor. Dr. Griffiths will continue his remarks on this subject at the November lecture at the club. Mrs. Fawcett's lectures on Problems of Poverty have commenced, and we noticed a few nurses amongst the audience, and many doctors. Mrs. Fawcett is an economist, and approaches the subject from that side; she speaks clearly and argues well.

COTTAGE NURSES.—Miss Broadwood sends us a letter pressing the needs of her system of cottage nurses. We are glad to see there is some talk of increasing the time of training from three to six months. Many of these cottage nurses are taught what they know at St. Mary's Mission, Plaistow, and they then go and work in country districts in Surrey and other southern counties. The immediate need of the group of humble associations working on this system, and employing between them 50 to 60 nurses (the number constantly increases), is a capital sum of about £1,000 to form a fund for teaching nurses, sufficient to pay out of its interest, the half-fees of a dozen pupils each year. Miss Pyne, of Westminster Hospital, is one of the supporters of this scheme.

MISS KATE MARSDEN.—It is a relief to those who appreciate the quiet work of a well-trained nurse to know that Miss Kate Marsden has reached Jerusalem, and commenced her investigation of the care of lepers without any preliminary fuss. Miss Kate Marsden is well known at Walsall, where she worked for many years, and also at Wellington, in New Zealand. She is an enthusiast, and has embarked on a great undertaking without cash and without fear. She is going to travel all through Siberia by the same route followed by the wretched Russian exiles; she will, for the next four or five months, be in daily peril. But Miss Kate Marsden has the Empress of Russia, the Princess of Wales, and Miss Florence Nightingale at her back, so that we look, and hope, for her safe return. Nurses will follow with interest the journeyings of this courageous member of their corps, and knowing that Miss Marsden holds that certificate of a true worker—a letter from Miss Nightingale—they will not need to fear sensational or ill-timed revelations or remarks.

HOPE HOSPITAL.—There is going to be a clean sweep at Hope Hospital apparently, and it is the very best thing that can happen. Evil associations hang about a place, and evil tales pass from tongue to tongue, and the knowledge of what has been, acts on what is. In cases of this sort it often happens that the innocent have to suffer with the guilty; for instance, every nurse within the hospital must feel very acutely the present unpleasant state of things. Most of the nurses who had to give evidence at the late enquiry have sent in their resignations, those who had not done so voluntarily will be requested to do so. In the meanwhile temporary medical aid has been procured for the patients. A well-known authority on union infirmaries declared the other day, that scandals were sure to be frequent, so long as there was no visiting medical staff—no direct communication, as it were, with the outer world. The question is worth considering. If changes are going to be made at Hope Hospital, they should be so thorough and startling as to utterly preclude any repetition of its past sad story, which time would soon blot out. All the teaching material which is wasted in these big infirmaries is a terrible loss, and if the utilising of it would also add to the better government of the internal affairs of the hospital, there is another reason for desiring the throwing open of the ward doors. There are 528 inmates of Hope Hospital just now, and many of the cases are serious; why then should not the neighbouring practitioners, or the specialists of Manchester grant their services to these sick, and have a hold, as it were, over the resident medical officers who will be appointed? Certainly nurses will be hard to get for Hope Hospital, unless some means are taken to remove the stigma which rests on its name.

### Sketches of Insanity.

By Francis H. Walmsley, M.D. (Senior Assistant Medical Officer of Leavesden Asylum).

(*Concluded from page* vi.)

### XIV.—MANAGEMENT OF THE INSANE.

"Adopt with every man the style and tone
Most courteous, most congenial, with his own."
—*Theognis.*

The cardinal principle underlying and directing all treatment of the insane may be briefly described as the endeavour to prevent the formation of a morbid habit of thought, and the prompt arrest of this brain habit where already formed. We have seen that a morbid self-feeling is a prominent characteristic of the insane, and one which displays itself in every stage of their malady. We must adopt such a course of rational treatment as will succeed in taking the patient fairly out of himself; efforts must be made to direct his thoughts and feelings from his morbid self-contemplation to that care and concern for others which is his normal state; those about him will endeavour to supplant his delusions and insane thoughts by other ideas, subjects, and occupations—as the latter gain a foothold and predominance, the former fade and disappear.

A man who lives a healthy, varied, natural life—who mixes freely with his fellow-men, who troubles himself much about their welfare and their happiness, who reads and thinks and works and plays, who vividly represents to himself the feelings and wishes and ideas of others—such a man as that seldom goes mad. He has no temptation. His surroundings are too sane and his interests too numerous. A family, friends, public duties, society—all these are safeguards against the insane tendency. Literature, science, art, politics—the wider your world the less your chance of nervous derangement.

It is often in vain to try to reason a man out of the indulgence of some master-passion, or the persistent pursuit of some favourite course of conduct; but excite some other passion or affection such, say, as ambition, or love of accumulation, or care for others, the passion which we wish to moderate or eradicate may with comparative ease be kept under control. Hence, to increase the intensity of any one passion or desire is to take an infallible means of weakening that of another.

The Commissioners in Lunacy have issued a valuable series of suggestions upon the nursing and management of the insane. They say: It is the duty of a good attendant to lead his patient, by advice and example, into habits of occupying and amusing himself; to encourage him when irritable; to supply his wants, however imperfectly expressed; to prevent all bad habits; and finally to bring him back gradually to the recollection of a former rational state, and stimulate his intellect when dormant, until it recovers its original tone and power.

Again, the study of the patient's character and peculiarities, the ascertainment of his fears and his delusions, his dreads and his suspicions; and then the dealing as tender-hearted, open-minded, sympathetic humane people with those mental conditions as mind to mind.

Every diversity and variety of influence must be brought to bear to remove as far as possible the cause of perverted feeling, to place the patient in a perfectly new surrounding of circumstances, and to trust, first, to the operation of time in wearing out morbid feeling, and, secondly, to the generation of feeling of an opposite and wholesome nature. Thus, nothing is more common than for an insane person to acquire antipathies to his dearest relatives and friends, accompanied or not by suspicions and delusions. So long as interviews with such relatives, or even intercourse by letter, or conversations about them with third persons, revive at intervals the full force of these feelings, no improvement takes place; but if the patient be removed from all contact with persons and things which suggest unhealthy reminiscences, if conversation respecting his morbid feelings is interdicted, and especially if all intercourse with the objects of these feelings is absolutely denied for a sufficient time, antipathy often gradually gives way to the yearnings of restored affection.

Finally, we should never forget we are dealing with fellow-beings who are insane, and in our relations with them should ever strive to establish a constant sympathising intercourse calculated to win their attachment and command their respect and confidence. So that, in the exquisite words of Ruskin, "It all comes at last to the forgetting yourselves and the living out of yourselves in the calm of the great world, or, if you will, in its agitation, but always in a calm of your own bringing. Do not think it wasted time to submit to any influence which may bring upon you any noble feeling. . . . You will love the creatures to whom you minister—your fellow-men. It is only kindness and tenderness which will ever enable you to see what beauty there is in the dark eyes that are sunk with weeping and in the paleness of those fixed faces which the world's adversity has compassed about till they shine in their patience like dying watchfires through twilight."

### Six Months in a Hospital.

(By a "Special Pro.")

### III.

Dearest C.—This is to show you that I have not forgotten you, although you evidently think it beneath your dignity to write to a poor little, hard-working nurse! I am beginning to get on a little better, I think; at least I feel a shade more used to it, but I feel sometimes as if I shall come back at the end of the six months as I first intended; but if I get over the strangeness I may like it well enough to stay. I am very much interested in the operations; and being a special "pro.," I am allowed, as a favour, to attend all I can be spared for. "Pro's," as a rule, do not go to any at first. One was amputation of the tongue for cancer; it was exceedingly interesting, and I was put on a few hours after as special nurse to watch the case for three hours. He had ice, and a little iced milk, but was fed for some time by nutritive enemas only, for fear of difficulties with the mouth. Another one was very dreadful. A poor old woman had had amputation of the leg above the knee, but the bone of the stump becoming "necrosed," it had to be recut off. It was sawn off, and actually the marrow scraped out and the bone syringed out. The poor old lady tried to sing in a funny little cracked voice under the chloroform. Another was operation for cleft palate on a sweet little child.

We have been singing hymns to-night in the W—— ward. The men were so delighted to have somebody to lead them, and a small boy, aged about seven, said he should like to sing all night!

I have lately been isolated with another diphtheria case, a very bad case too, "typical" diphtheria. They performed tracheotomy as a last chance for him, two hours after I had gone off duty, but he died immediately. My other case was very sad. I don't know whether I told you. A little boy was brought in very bad, and died the next day. A day or two after his sister, a baby girl, was brought in with the same thing. They "trachyd" her to no purpose. The poor mother was nearly wild. They were her only children. The worst of it is, so many are caused simply by the ignorance or wilful carelessness of people, who, if they are not intentionally villains, must be simply fools! This boy of 17, my last case, was an instance of this. Three of the children were taken ill, two recovered more or less, this one died; and now they find an open drain, untrapped, put in a short time ago, just beneath the boy's window! All night long the exhalations came into his room, and of course the end was diphtheria. Our modern civilisation, indeed!

It is rather jolly being isolated; I have written my diary, tied a lot of cap strings into bewitching little bows, and am now writing letters. You see in an isolated ward you are "boss" (forgive the slang), and you can do as you like. I see the doctors, make out the report, get the medicines, carbolic acid, &c., from the dispensary, and give the night nurse her instructions, if there are any. So you see I feel quite grand! I must tell you that I went up for my physiology examination at —— last Friday. I answered all the questions after a fashion—examiners are Professor Huxley and Dr. M. Foster. I felt inclined to turn and come home again when I saw who they were!

I am in a surgical ward now, and such a nice one. We have some very interesting cases in. One a sweet little girl, run over by a Great Northern Railway van; broken one arm and one leg. She is getting on splendidly, and says she loves us so much, that she does not want to go home again. I am so fond of her that I really feel sorry to think of losing her. Another is a charming little girl with hip disease, and another with the same complaint is a dear little boy of four years. He told me to-day that his father is very fond of him, but beats his "mummy," and puts the poker in the fire to burn her with; and when he was going to burn her one day, "granny" wouldn't let him, and they all fought, and then the policeman came, and then he said "father beat her till the blood all came round her like this," and he rubbed his little hands all over his face. What a home! and he looks like a little cherub, with gold curls, and a lovely pink and white complexion. Isn't it sad to think of the transformation scene that will take place in him in a few more years? What can anyone expect of the children when their earliest recollections are such as these!

*(To be continued.)*

### Southall's Patents.

All nurses know by this time the tremendous advantage of using in nursing, and at all times, Southall's sanitary towels for ladies; for comfort and cleanliness they are unrivalled, and they decrease risk of infection or chill. Monthly nurses would do well to acquaint themselves with the advantages of the infant's "Knapkenette," which is of great use in preventing soreness and chafing. The "Kenilworth" Violet Powder, manufactured by Southall Bros., deserves mention, for it is deliciously soft and smooth, and equally suitable for the nursery or the toilet. The "Combination Suspender" is another patent, which, from its bearings on health and comfort, deserves the attention of nurses (but could not it be made of wool, and thus lessen the risk of chill?) and the sanitary sheets for accouchements are surely appreciated and known to every midwife and nurse who keeps up with the times, and is interested in her profession. One great point about Messrs. Southall's preparations is their cheapness.

### Death in Our Ranks.

Nurse Constance Beckles, of the Ormskirk Union, died at her post on the morning of October 6th. She was only twenty-five years of age, and a very bright and cheerful worker; she had mentioned to the doctor that she would like to die on duty. Nurse Beckles will be a great loss to the ranks of the Mary Adelaide nurses. She died of heart disease.

### The Nurses' Bookshelf.

### ADVICE TO MOTHERS.*

Is it true that lookers on see most of the game? If so, it will account for the excellence of this handbook, for unless we are much mistaken the authors of "Our Nurses and the Work they have to do" are not greatly experienced in the subjects they treat. To begin with, this is not a scientific book; it is written in a popular strain and is full of sentiment. It tells as much about the soul as about the body; there is a great deal about typical motherhood, our mother-church, and Heaven's reflections. But mixed up with all these digressions are very plain and very useful hints for young mothers, which are so essentially practical they make the digressions appear more extraordinary. There is a certain class of nervous, shallow, sentimental women, to whom this book would appeal, and it would perhaps comfort them better than a simple set of rules which did not include treatment for the soul. The authors write in a chatty manner which is easy to read, their language is simple; every paragraph is side-headed; it requires no intellect to comprehend what is here set down, only we doubt if a mother ought to be so childish as to need this commonplace good advice. Granted that is necessary, then we grant the practical part is excellent, though the sentiment seems to us rather thin, and the religion decidedly crude. There is a full index to the book where you will find side by side "Gifts of God," "Glands, swelled"; or "Spiritual Preparation," "Spurious Pains;" fair proof of the strange mixture of common-sense and sentiment of which the book consists.

### NURSERY HEALTH TRACTS.*

These small tracts are simple yet conci se, and will be invaluable to district nurses. The information given is complete in its way, and will teach many a nurse points on which she is ignorant, but at the same time the tracts are eminently suited for public distribution. There is nothing in them every mother ought not to know. The tracts are very daintily got up, but we should have preferred, if it were possible, that their price should be a penny; they ought to be within the reach of the poorest. Scarlet Fever is treated by John M. Keating, M.D., of Philadelphia; Diet for Young Children by Dr. Holt, of New York; Diphtheria by Dr. H. Dwight Chapin, of New York; and Sound Teeth for Children by Dr. Freeland D. Leslie. The tract on scarlet fever we should like to see very widely distributed; the tract on teeth contains the most unusual information.

* "New Life; Its Genesis and Culture." By H. C. O'Neil and Edith A. Barnett. (London: Messrs. Swan Sonenschein and Co.)

* "Diphtheria," "Scarlet Fever," "Diet for Young Children," "Sound Teeth for Children." 2d. each. (Babyhood Publishing Company.)

### Examination Questions.

Once more with the short days we commence our competition in the answering of examination questions, and thus hope to help young probationers to pass that dreaded ordeal which leads to a certificate. The question for this month is—"What precautions for her own safety, and for the safety of others, should a nurse take while nursing a case of typhoid fever?" Answers must be addressed to the Editor, the word "Nursing" being written at top of the envelope, and must reach this office by November 1st. Answers must be short and concise, and written on one side of the paper only; the writer must send her full name and address. The best answer will be printed in these pages, and a book will be presented to the writer. All nurses may enter for this competition.

### Everybody's Opinion.

THE NURSES OF THE LONDON HOSPITAL.

"D. C." writes: It is wonderful how easily people, who are probably well meaning enough in their way, allow themselves to fall into errors when discussing really important matters. In the great controversy regarding the London Hospital and its nursing department, an attempt was made to show that the matron sent out insufficiently trained nurses to perform private work. The basis of the argument was that inasmuch as probationers on entering the hospital are all bound for a period of two years, therefore they are not proficient before the expiration of that period, and therefore if sent out to private cases after completing but one year's training, as is the custom, they are insufficiently trained. Now, as a matter of fact the period of apprenticeship, or whatever it may be called, has no connection whatever with the time that the authorities consider it necessary a nurse should train to gain proficiency. One years' training is believed to be long enough for this. The object of a two year's agreement is to ensure that the nurse will not leave the institution after gaining her certificate, and so deprive it of her services, after it has been to the expense of teaching her. All the hospitals have their own rules regarding the period which nurses bind themselves to serve. For instance, St. Mary's imposes three years' services, and so on. This variation alone is sufficient evidence that the term of apprenticeship has nothing to do with the time necessary to gain proficiency. So there is no foundation for the accusation that Miss Lückes sends out nurses to private cases before they are qualified to perform their duties. Neither is there the smallest foundation for the statement, made, it appears, by a medical man—who is evidently not a business one, however—that nurses go out and earn £1,200 a-year for the hospital, and are "sweated" by being paid a miserable of say £16 per annum, the institution pocketing the rest, like an avaricious East-end tailor. The fact is that nurses on private work at the London get £28 per annum, and their board and lodging, and in return go where the authorities order them. It is true that their wages may repay the hospital, or even bring it a profit. But there are features in the contract that deserve notice: (1) The nurse gains by having a regular guaranteed salary; (2) were she practising on her own account she would receive but a fraction of the cases, these coming in by reason of the "connection" which a large institution like the London naturally has; (3) in the intervals between her private cases she has the run of the hospital, where she can renew her training and keep her knowledge up to date; (4) as a servant of a hospital, her character, status and integrity are established, and beyond challenge. But of all the advantages the first is the greatest, viz., certainty of income; for nine women out of ten, and ninety men out of a hundred would give up the whole of a chance income to secure a certainty half its size.

### Appointments.

[It is requested that successful candidates will send a copy of their applications and testimonials, with date of election, to The Editor, The Lodge, Porchester Square, W.]

Levick Institution, Paris.—Nurse Pyne, who has just completed her training at the Hospital for Sick Children in Great Ormond Street, has been appointed Assistant Superintendent of this Institution.

Seamen's Hospital, Greenwich.—Miss E. B. Fraser, late head nurse at the Norfolk and Norwich Hospital, has been appointed Sister of the Somerset Ward of this Hospital out of 81 candidates.

Barnhill Poorhouse.—Miss Sarah Ellen Clarke, who trained at the Bradford Nurses' Institution, and has since worked at the Croydon Institute, has been appointed Superintendent of the Children's Department of this Poorhouse.

### Notes and Queries.

### Queries.

M. S. would be greatly obliged if the editor of The Hospital could recommend her an inexpensive manual on drainage, ventilation, &c., suitable for a lady going in for an examination following a course of health lectures.

### Answers.

*Queen's Jubilee Institute.*—We beg to inform Mrs. Ratcliffe that there is a branch of this institute in Edinburgh, and they will train her the district nurse she desires. All queries regarding the Jubilee Institute should be addressed to the Hon. Sec., at St. Katherine's Hospital, Regent's Park, N.W., or particulars will be found in *The Hospital*, for July 12th, 1890.

*A District Nurse.*—Many thanks for your interesting notes which we will publish next month.

*Nurse Addie.*—The verses are too similar to some which we have already in type.

*F. M. B.*—You must send name and address, not for publication, but as evidence of good faith, before we can print your letter.

*Vanessa* wishes to thank those who have answered her query, and regrets she cannot work for all the institutions she has heard from.

*J. M., Birmingham.*—We do not know the purpose to which it is desired to put the apparatus, but we think J. Taylor and Sons Portable W.C. would meet your requirements.

*M. S.* will find "Helps to Health" (Kegan Paul, Trench, and Co.) just the manual for her purpose. It is most suitable for a lady going in for an examination after a course of health lectures.

*Miss Hale, Rotherham Hospital*, would like to hear at once from all those of the "First Thousand" to whom she wrote in August, and who have not yet replied to her circular.

### Lectures and Amusements.

St. John's Ambulance Association.—A women's "First Aid" class will commence on October 28th, at quarter-past six p.m., at St. John's Gate, Clerkenwell; a men's "First Aid" class on October 28th, at eight p.m. Fee 7s. 6d.

Welbeck Hall.—Mrs. Fawcett commenced a course of lectures on October 10th, at half-past eight p.m. (to be continued on following Fridays), on "The Problems of Poverty." Admission 1s. each lecture. This course will also be given at Highgate High School, commencing Oct. 29th.

Toynbee Hall.—On October 18th, at eight p.m., Dr. Albert Leffingwell, of New York, will lecture on "How to Die of Old Age."

St. George's Hall.—Mr. and Mrs. German Reed's entertainment is given on Mondays, Wednesdays, and Fridays, at eight p.m.; on other days at three p.m. Nurses will find the 2s. seats excellent, if they get there in good time. Mr. Corney Grain's new sketch is delightful.

### Vacancies.

The following vacancies are announced:

### Nurses.

Aberdeen Fever Hospital; £26.
Barony Parochial Hospital, Glasgow; £24.
Bedfordshire Hospital Institute.
Bristol Infirmary; £26.
Bristol Hospital for Children and Women.
Bromley Institution.
Bournemouth Institution; £25.
Borough Hospital, Birkenhead.
Bath Home.
Burton-on-Trent Intitution; £25.
Blackheath and Richmond Institutions.
City of London Chest Hospital; £20.
City Hospital, Coventry (Assist.).
Easter Ross Combination Poor House; £30.
General Nursing Institute, Covent Garden.
Hampshire Institute, Southampton.
Hereford Infirmary; £20.
Hull Home.
Hanover Institute; £25.
Jersey Institute, £25.
Jessop Hospital, Sheffield; £25.
Kensington Infirmary; £20.
Liverpool City Hospital (Charge); £35; also Assist.; £25.
London Ophthalmic Hospital.
Middlesborough Association.
North-Western Fever Hospital; £30.
Norwich Home.
Nurses' Home, Bengeo, Herts; £30.
Nurses' Home, Sheffield; £25.
Nottingham and Notts Institution; £25.
Rochdale Infirmary; £22.
Royal Albert Hospital, Devonport; £25.
Rural Nursing Association.
Sheffield Fever Hospital; £25.
Samaritan Free Hospital, N.W.
Southport Infirmary; £26.
St. Mary Abbott's, Kensington, Infirmary (Supt.); £30.
Suffolk General Hospital, Bury St. Edmunds; £20.
St. Leonard's, Shoreditch, Infirmary; £20. (also Assist., £17).
Smithston Hospital, Greenock; £25.
St. Albans Hospital, Herts.
St. Saviour's Infirmary, East Dulwich.
St. Helena Home.
Victoria Home, Bournemouth; £25.
Whitechapel Infirmary; £20.
Wilson Institution.
Wirrall Children's Hospital (Head).
Workhouse Infirmary Association.

### District Nurses.

Bristol Society; £42.
Gt. Bedwyn, Wilts; £25.

### Probationers.

Bolton Infirmary.
Boston Hospital, Lincolnshire.
Horton Infirmary, Banbury.
Leeds Fever Hospital.
Leeds Institution; £14.
Suffolk General Hospital, Bury St. Edmunds.
St. Helena Home.
Weston-super-Mare Hospital.
Workhouse Infirmary Association.

### Matrons.

James Murray's Royal Asylum, Perth.
Deptford Crêche.
Lowestoft Hospital; £50.

### Sisters.

National Hospital for the Paralysed and Epileptic; £30.

Bolton Infirmary.

### Amusements and Relaxation.

**N.B.—THIRD QUARTERLY WORD COMPETITION**

**Commenced Oct. 4, 1890; ends Dec. 27, 1890.**

Three prizes of 15s., 10s., 5s., will be given for the largest number of words derived from the words set for dissection.

The word for dissection for this, the THIRD week of the quarter, being "MEDICINE."

| Names. | Oct. 11th. | Totals. | Names. | Oct. 11th. | Totals. |
|---|---|---|---|---|---|
| Jenny Wren | 55 | 55 | Sydenham | 38 | 38 |
| Tinie | 55 | 55 | Liz | 38 | 38 |
| Agamemnon | 55 | 55 | Checkmate | 33 | 33 |
| Patience | 55 | 55 | Silver King | 33 | 33 |
| Ecila | 53 | 53 | S. Anthony | 33 | 33 |
| Lightowlers | 52 | 52 | Quackah | 31 | 31 |
| Rouge | 52 | 52 | Reynard | 30 | 30 |
| Wyamaris | 45 | 45 | Sally | 27 | 27 |
| Qu'appelle | 45 | 45 | Success | 26 | 26 |
| Nosam | 45 | 45 | Caledonia | 26 | 26 |
| Nurse Hilda | 44 | 44 | Nurse Emma | 25 | 25 |
| Lady Betty | 43 | 43 | Hazel | 20 | 20 |
| Grenelle | 43 | 43 | Pallas | 19 | 19 |
| Daisy | 40 | 40 | Puss | 15 | 15 |
| H. A. S. | 39 | 39 | | | |

